# The most common nursing diagnosis among adults/seniors hospitalised with cancer: integrative review

**DOI:** 10.3332/ecancer.2014.462

**Published:** 2014-09-03

**Authors:** Rafael Tavares Jomar, Vitória Régia de Souza Bispo

**Affiliations:** Instituto Nacional de Câncer José Alencar Gomes da Silva, Rio de Janeiro 20230-130, Brazil

**Keywords:** nursing process, nursing diagnosis, oncologic nursing, hospital oncology service

## Abstract

The nursing process, with emphasis on the diagnosis phase, is essential to oncology hospital services due to a high frequency of physical and psychological problems that compromise the quality of life of patients undergoing cancer treatment. The goal of this study was to identify, according to NANDA International, the most common nursing diagnosis among adults/seniors with cancer who are hospitalised. This study is an integrative review of the literature completed in 2013 using five electronic databases, resulting in the selection and analysis of nine articles. This review identified the following eight actual diagnoses and two risk diagnoses that are more common among hospitalised adults/seniors with cancer: anxiety, deficient knowledge, constipation, self-care deficit for bathing/hygiene, body image disturbance, acute/chronic pain, fear, disturbed sleep pattern, risk of infection, and risk of deficient fluid volume. The heterogeneity of the studies used in this review may not have allowed the identification of all the common nursing diagnoses in the practice of oncology nursing in hospitals. However, even though the results are not based on the highest possible level of scientific evidence, their correlation to clinical practice can contribute to the enhancement of the nursing process in oncology services provided by hospitals.

## Introduction

The nursing process is the orienting method that guides the actions of nurses in daily professional practice, and it offers a structure that is concordant with the individual needs of the patient, the family, and of the community, representing the main methodological instrument for the systematic performance of the necessary conditions to provide care and documentation in nursing practice [1].

The nursing process has five steps: data collection, diagnosis, planning, implementation, and evaluation. In theory, these phases are limited; however, in practice, they represent a group of interdependent actions and then, in turn, when evaluating the patient, hypotheses emerge and lead to diagnoses that determine the care, which will be implemented and re-evaluated [2].

In the present study, one phase of the nursing process stands out: the nursing diagnosis (ND). Defined as a clinical judgment about the client’s, the family’s, or the community’s responses to health problems/vital, actual, or potential processes, the ND provides the basis for the selection of nursing interventions aiming to achieve the results for which the nurse is responsible [3].

In this phase, the nurse must analyse the collected data during the assessment and evaluate the health condition of the patient. In a clinical reasoning process, needs are identified from the interpretation and grouping of the collected data. Some of the conclusions resulting from this process will lead to the ND, others will not [4]. Once an ND is inferred, an outcome to be reached is determined and a double commitment is created: to intervene and, subsequently, to evaluate, the efficacy of the performed intervention [5].

The use of the ND has brought some advantages to the practice, such as the holistic approach to the patient, the acquisition of a proprietary body of knowledge, the search for improvement in the quality of the service provided, and the fostering of continuous improvement of nurses [6]. Moreover, when using the nursing process, nurses begin to gain more information on which to base their interventions, since the ND is considered to be the guide for the selection of the most adequate interventions to achieve the desired outcomes for each individual in the context of care [7].

In light of this, it is believed that, in oncology hospital services, the nursing process with emphasis on the diagnosis phase is essential to the oncology hospital services due to a high frequency of physical and psychological problems that compromise the quality of life of patients undergoing cancer treatment.

Cancer represents more than physical pain and discomfort. It affects the patient’s life goals, family, work, and income. His/her mobility, body image, and lifestyle can be temporarily or permanently drastically altered [8]. Therefore, the nurse has a great responsibility in the planning of nursing assistance in oncology, especially regarding decision-making and actions oriented to solving the problems identified in the diagnostic phase of the process.

In Brazil, cancer is considered to be the second most common cause of death, and approximately 576,580 new cases of the disease are expected for the year 2014. The most common types will be melanoma (182,000), prostate cancer (69,000), breast cancer (57,000), cancer of the colon and rectum (33,000), lung cancer (27,000) and stomach cancer (20,000) [9].

In light of these estimations, and knowing that, frequently, cancer patients need to be hospitalised to receive treatment, nurses working in oncology hospital services must provide care focusing on the needs of the individual, using the ND as a tool for the standardised identification of outcomes aiming to achieve or maintain the best health condition of patients [10].

Considering the lack of publications on the ND in oncology and the fact that the knowledge of the most common NDs in this field can strengthen the nursing process and provide more information on which oncology nurses can base their decision-making actions, the selection of the best interventions and the performance of a competent clinical practice, the goal of this study is to identify the most common NDs among adults/ seniors hospitalised with cancer.

## Methods

This study is an integrative literature review that aims to group, analyse, and summarise the results of research on a pre-determined topic, in a systematic and ordered way, contributing to the enrichment of the researched theme. The integrative review is one of the methods applied to the evidence-based practice that allows the incorporation of research evidence to the clinical practice [11].

In order to write this review, the following steps were taken: formulation of the research question, literature search, categorisation of research articles, evaluation of the articles included in the review, discussion and interpretation of the research findings, and the synthesis of knowledge evidenced by the analysed literature [11].

The research question of the present review is the following: What is the most common nursing diagnosis among adults/seniors hospitalised with cancer?

In October of 2013, with no restrictions to the publication date, the nature of the research or language, the two authors of this review independently proceeded to search for research articles indexed in the electronic databases Medical Literature Analysis and Retrieval System Online (MEDLINE), Cumulative Index to Nursing and Allied Health (CINAHL), Literatura Latino-Americana e do Caribe em Ciências da Saúde (LILACS), Scientific Electronic Library Online (SciELO), and Base de Dados de Enfermagem (BDENF). To find the research articles, the keywords *Enfermagem Oncológica/Oncologic Nursing* and *Neoplasias/Neoplasms* were each combined with the keywords *Diagnóstico de Enfermagem/Nursing Diagnosis*.

The inclusion criteria used in the selection of the studies for this review are the following: the ones published in the format of an original article in English, Spanish, or Portuguese and which described quantitatively, in their findings, the ND identified in such population according to NANDA’s International taxonomies I and II [3]. The exclusion criteria adopted were: literature reviews, updates, case/experience reports or any other format in which the findings were not described as being obtained from collecting data (interviews and/or physical exams and medical records) from hospitalised cancer patients.

It is pertinent to highlight that the eligibility criteria adopted are based on the fact that the main evidence that supports clinical practice corresponds to the findings of the research [12].

In the databases, 86 articles were found on MEDLINE, 12 on CINAHL, 119 on LILACS, three on SciELO, and 165 on BDENF, totalling 385 studies. According to the goal of this integrative review, the articles were pre-selected by title and summary, according to the inclusion and exclusion criteria adopted. In the databases, search articles were pre-selected as follows: eight articles from MEDLINE, one from CINAHL, nine from LILACS, none from SciELO, and seven from BDNENF, totalising 25 articles. After the removal of duplicates and the full reading of the studies, the final sample had nine original articles. The two authors performed the process described above and a third collaborator mediated the disagreements.

To extract the information from the selected articles for the review, a data collection form was developed, in which the NDs that represented a frequency equal to or greater than 20% was registered, meaning that, in each study, only the NDs with high magnitude were extracted. The form allowed the acquisition of information about the article’s authors, country and year of publication, source localisation, goals, research design and characteristics, data analysis, findings and discussion, and conclusion and recommendations for nursing practice.

Afterwards, the NDs extracted in the prior step that belonged to at least three of the nine selected articles were considered to be the most common among adults/seniors hospitalised with cancer. That is, it was assumed that the nine selected articles (100%) are the units of analysis of the present review and that the extracted NDs with a prevalence of approximately 30%, i.e., present in at least three of them, are the most frequently found/described in such population, even if in distinct magnitudes.

The analysis and synthesis of the articles were performed descriptively to evaluate the quality of the evidence, according to the criteria established by nursing scholars [13].

## Results

From the nine original articles included in these integrative reviews, four were published in the English language and five in the Portuguese language. In terms of the countries of origin among the studies, Brazil stands out with five articles. In relation to the academic journals, the articles were predominantly published in the *International Journal of Nursing Terminologies and Classifications* (formerly *Nursing Diagnosis*), which published three articles. Regarding the year of publication, four articles were published in the 1990s, four in the 2000s, and one in 2013.

As for the source of research data, five articles used medical records, three articles used interviews and physical exams, and only one used all of the above-mentioned sources. Regarding the NANDA International [3] Taxonomies, four articles, published until 1998, used Taxonomy I, and five articles, published since 2005, used Taxonomy II. Three articles did not directly publish the value of the relative frequency of the NDs in the studied population, which was calculated using the absolute numbers of NDs and participants.

A synthesis of the articles included in the present integrative review is described in Table 1. As all of the reviewed studies have a descriptive nature, it is worth highlighting that they generated a low quality level of evidence: level 6, on a scale of 1 to 7, in which level 1 represents the highest degree of evidence quality [13].

The most common NDs among hospitalised adults/seniors with cancer, that is, those who, after the identification of frequencies greater than or equal to 20% in each of the articles selected, identified in at least three of the articles included in this review (prevalence of 30% in the review), are described in Table 2.

## Discussion

The present integrative review identified eight actual NDs and two risk NDs that are more common among adults/seniors hospitalised with cancer, as follows: anxiety, knowledge deficit, constipation, self-care deficit for bathing/hygiene, disturbed body image, acute/chronic pain, fear, disturbed sleep pattern, risk of infection, and risk of fluid volume deficit.

The risk of infection ND was described in high frequency in all of the studies included in this review, except for one, due to its own goal, which was to identify only the emotional NDs. The risk of infection is defined as the state in which the patient is at risk of being invaded by an opportunistic or pathogenic agent of endogenous or exogenous sources and has the cancer as one of its risk factors, as well as the radiotherapy, chemotherapy, surgery, bone marrow transplant, immunosuppression, and hematologic disturbances [4]; treatment modalities and arising problems common in hospitalised cancer patients.

The risk of fluid volume deficits ND is defined as a state in which the patient is at risk of presenting vascular, interstitial, or intracellular dehydration and has, as related factors, among others, the excessive loss of liquid by drains, described in one of the studies included in this revision [16]. Another study included here [15] points out that a reasonable explanation for the presence of this ND among hospitalised patients with cancer is the complexity of their physiologic state, disturbed by the neoplasia or its treatment. For that reason, patients with acute myeloid leukaemia should have this ND investigated, due to the use of a variety of medicines, electrolytic disturbances and the excessive elimination of liquid (consequent to the increased frequency of vomits and diarrhoea) is greater than the ingestion of liquids [19].

The Pain ND is defined as a state in which the patient presents and reports serious discomfort or uncomfortable sensation for under six months (Acute Pain) or for longer than 6 months (Chronic Pain) [4]. It was reported with expressive frequencies in more than half of the studies included in this review, either among the clinical [14, 15, 17, 19] and surgical [20] patients, or among the ones at the end stage of their life [18].

A meta-analysis study [23] points to the increased prevalence of pain in cancer patients: 53% of patients in any phase of the treatment, 64% among the ones with metastasis, advanced disease or terminal, 59% among the ones in antineoplastic treatment, and 33% among cured patients.

Emotional state, former painful experiences, and cultural factors influence the way in which a patient responds to pain. Feelings such as angst, fear, and exhaustion related to the hospitalisation and the disease itself worsen the patient’s reaction to the sensation of pain. As a result, not only the systematic investigation of the ND acute and/or chronic pain is shown to be imperative, but also the evaluation of the nursing interventions performed, which should satisfactorily relieve the experience of pain of the hospitalised cancer patient, so that it does not increase even more the discomfort already caused by the hospitalisation.

It is pertinent to highlight the importance of monitoring the side effects of the opiates that are commonly used for the treatment of the pain in cancer patients, such as constipation, nausea, and vomiting. Constipation, defined as the state in which the patient presents large intestine stasis, resulting in infrequent bowel movement and/or hard and dry stools [4], was also one of the NDs characterised by this present review as being very frequent among adults/seniors hospitalised with cancer, possibly because of the regular administration of opiates for pain management.

In this context, the pain directly interferes with the comfort of the patient, affecting his/her nutrition, daily activities, and sleep patterns [20]. This review also characterised the disturbed sleep patterns ND as one of the more frequent among adults/seniors hospitalised with cancer. This ND is defined as the state in which the patient present a change in the quantity and quality of his/her rest pattern, causing discomfort, or interfering with the patient’s desired life style [4]. Because the sleep pattern has, in addition to pain and anxiety, the effects of hospitalisation on sleep as related factors [4], it was expected that this study would characterise such an ND as one of the more frequent ones.

The anxiety ND is defined as the state in which the patient presents feelings of unrest and activation of the autonomous nervous system in response to a vague and unspecified threat. In turn, the Fear ND is defined as a state in which the patient presents a feeling of physiological or emotional disturbance related to an identifiable source, perceived as dangerous [4].

The three studies [19, 20, 22] included in this review that presented the Fear ND also presented the Anxiety ND. The validation study of the Anxiety and Fear NDs [24] highlighted the presence of defining characteristics and related factors, similar to these diagnoses, because they share 20 equal or similar defining characteristics, which makes it difficult to differentiate between these human reactions. One of the studies included herein [22] reports that these NDs were commonly recorded in medical records by nurses under *Anxiety/Fear*.

Many factors can cause anxiety in hospitalised cancer patients, such as worries about the surgery outcomes, being for either the diagnosis confirmation or the removal of body parts; factors that generate fear, in general, are related to pain and to chemotherapy. Moreover, these feelings can affect the patient’s ability to absorb important information about hospitalisation, thereby feeding the knowledge deficit [20].

The Knowledge Deficit ND, which is defined as the state in which the patient presents a cognitive knowledge deficit or psychometric abilities deficit related to the health condition or to the treatment plan [4], was also one of the NDs characterised by this review as one of the more frequent among hospitalised adults/seniors cancer patients. What stands out, however, is the fact that three of the five studies that identified such an ND did not specify what topics represented the patients’ information deficit.

The Disturbed Body Image ND is defined as the state in which the patient presents a rupture in the way he/she perceives his/her body image and has as related factors, among others, changes in personal appearance secondary to surgery, radiotherapy and chemotherapy [4], treatment modalities more common to hospitalised cancer patients that may lead to changes and, even more, the loss of body parts.

In addition to alopecia, the most common side effect of chemotherapy treatment, there are other factors that can contribute to the presence of a Disturbed Body Image among cancer patients, such as weight loss, weakness, paleness, and hematoma.

Bathing/Hygiene Self-Care Deficit ND is defined as the state in which the patient presents impaired ability to perform or complete bathing/ hygiene activities. The identification of this ND was already expected as one of the more frequent NDs due to the multiple reasons that lead adults/seniors with cancer to become hospitalised. This is because surgery, chemotherapy, radiotherapy, management of serious symptoms, and adverse reactions to the treatment may lead to a dependency on the nursing care practices related to bathing/hygiene.

None of the studies included in this review that had adult/senior participants pointed to the differences in the distribution of the NDs among these populations. When comparing the studies’ findings that had adult participants with the ones that only had seniors, the same was observed: there seems to be no difference in the distribution of NDs among these populations. Hence, we recommend that studies on the evaluation of the possible differences in the distribution of NDs among adults and seniors be conducted in the future, since the aging process is one of the more important factors in the oncogenesis process [9], which could suggest the differences in the distribution of NDs.

The intellectual process in the formulation of an ND requires objectivity, critical thinking, and decision-making. Therefore, the diagnostic process implies a systematic and in depth analysis of the affected patient’s basic needs which the nurse makes by carrying out a planned, critical, and scientific approach [6].

However, some studies included in this review had only the medical records as the source of the data, which does not seem to be enough to obtain information about defining characteristics of NDs. One of the studies included in this review affirms that additional methods of data collection, such as interviews and physical exams, can be useful to minimise this bias.

The limitations of this review pointed out the great variability of the small sample sizes of the studies included herein, as well as the lack of statistical treatment of the data from each study. Another limitation that deserves to be mentioned is the different characteristics of the studies included in it: three studies had only female subjects [16, 18, 22], one had only male subjects [21]; one study aimed at identifying only emotional ND [20], another one emphasised the identification of psychosocial and spiritual ND [22]; two studies elected the postoperative phase for data collection [21, 22], one elected the preoperative phase [20], another one elected the time of hospital admission [16], and another the time of discharge [14].

Thus, the heterogeneity of the components of this review studies may not have allowed the identification of the most common and high-frequency NDs in hospital nursing practice in oncology, such as Fatigue, for example. It is noteworthy, however, that even if the characteristics of the patients and the magnitude of NDs described are different in the articles, the adults/elderly participants in the study, regardless of medical diagnosis, treatment modality, and reason for hospitalisation, have in common the fact of being hospitalised for the treatment of cancer, allowing generalisations for such a population.

Therefore, if the need for publications that address NDs in the daily practice of oncology nursing is considered, considering the scarcity of publications on the subject, the results of this review provide a more general mapping of common NDs in adults/elderly people hospitalised with cancer, thus contributing to improve the nursing process in hospital oncology services.

## Conclusions

From the analysis of nine studies, this integrative review identified ten of the most common NDs in adults/elderly hospitalised with cancer, namely: anxiety, knowledge deficiency, constipation, self-care deficit for bathing/hygiene, body image disturbance, acute/chronic pain, fear, disturbed sleep pattern, risk of infection and risk of fluid volume deficiency.

Although the NDs identified are not based on highly categorised scientific evidence by the current model, and therefore do not constitute irrefutable recommendations, it is important to link the knowledge derived from these studies to clinical nursing practice.

## Conflicts of Interest

The authors have no conflicts of interest to declare.

## Contributions of the authors

RTJ participated in the conception and design of the article, literature review, analysis and interpretation of data, discussion of results, drafting and final approval of the article.

RVSB participated in the literature search, critical revision and final approval of the article.

## Figures and Tables

**Table 1.** Summary of the articles included in the integrative review.

Author, country, and year of publication	Design, source, sample, and quality level of the evidence	Objective(s)	Main findings	Recommendations for the nursing practice
Sheppard [14], US, 1993	Descriptive (Cross-sectional) Medical Records *n* = 196 Evidence level 6	To identify the nursing diagnoses in lung cancer patients at the time of discharge from different health services and to describe the complexity of the necessary care in the community	Among the 16 hospitalised patients and according to NANDA’s International Taxonomy I: Constipation (63%), Impaired physical mobility (63%), Risk of Infection (63%), Self-Care Deficit for Bathing/Hygiene (56%), Impaired Gas Exchange (44%), and Pain (44%). Longer hospitalisation time and better socioeconomic status of the patients resulted in an increased need for care in the community.	The nursing diagnoses do not only constitute theoretical basis for the identification of phenomena of concern to nursing but are useful in clinical practice, helping nurses to predict the needs for resources in the community after the hospital discharge. Therefore, from the time of hospital admission, nurses must already identify the predictors of high-risk nursing care needs at the moment of the discharge of cancer patients.
Chang *et al* [15], US, 1995	Descriptive (Cross-sectional) Physical Exams and Interviews *n* = 59 Evidence level 6	To identify nursing diagnoses in hospitalised seniors with cancer and determine if the ones with history of allergies were at risk for the selected nursing diagnostics	According to NANDA’s International Taxonomy I, among all of the seniors: Pain (58.6%), Risk of Infection (47.4%) and Impaired Integrity of the Skin (27.1%). Seniors with history of allergy were significantly more prone to Risk of Infection (90.6%) than the ones without history of allergy (51.8%). Knowledge Deficit [not specified] (25%) and Risk of Deficient Fluid Volume (21.9%) occurred in a significant number in the group of seniors with history of allergy.	The identification of immunologic deficiencies is useful to guide the nursing care provided to the senior with cancer, particularly among the ones with allergy history. The nurse evaluation must include the nursing diagnoses associated with immunological deficits (Potential for Infection and Potential for Deficit of Fluid Volume), as well as protection factors (integrity of the skin and of the mucous membranes). Greater attention must be directed to the factors that increase the immunity, as such as nutrition, adequate hydration, skin care, and stressors reduction.
Lopes *et al* [16], Brazil, 1997	Descriptive (Cross-sectional) Medical Records *n* = 30 Evidence level 6	To identify nursing diagnoses more frequent in women in the moment of hospital admission when submitted to oncologic surgery	According to NANDA’s International Taxonomy I: Risk of Infection (100%), Risk of Imbalance Body Temperature (60%), Risk of Aspiration (36.6%), Colonic Constipation (26.6%), Risk of Impaired Physical Mobility (26.6%), Potential for Altered Protection (23.3%), Risk of Deficient Fluid Volume (20%).	Nursing diagnosis opens possibilities for the development of nursing because it creates its own language to describe the patient problems that the nurse has competence to solve. The routine use of nursing diagnoses would contribute to better define the clinical practice of the oncologic nursing, as it is a complex specialty.
Courtens and Abu-Saad [17], Holanda, 1998	Descriptive (Cross-sectional) Medical Records *n* = 15 Evidence level 6	To identify nursing diagnoses, their defining characteristics and factors related to leukaemia hospitalised patients	According to NANDA’s International Taxonomy I: Disturbed Sleeping Pattern (93.3%), Fatigue (86.7%), Risk of Bleeding (86.7%), Impaired Skin Integrity (86.7%), Excessive Fluid Volume (86.7%), Pain (80%), Imbalanced nutrition: less than body requirements (80%), Impaired oral mucous membrane (80%), Nausea (73.3%), Risk of Infection (66.6%), Diarrhoea (66.6%), Bathing/hygiene Self-Care Deficit (66.6%), Hyperthermia (60%), Vomit (53.3%), Physical Mobility Impaired (46.6%), Knowledge Deficit [diagnostic, treatment, and Isolation] (40%), Impaired Swallowing (33.3%), Ineffective Breathing Pattern (33.3%), Ineffective oping (33.3%), Impaired Social Interaction (33.3%), Dizziness (26.6%), Pruritus (26.6%), Urinary Incontinence (26.6%), Risk of Fluid Volume Deficit (20%), Recreational Activity Deficit, Hopelessness (20%), Ineffective Family coping (20%)	Nursing care for leukaemia patients is very complex and different functional health standards are affected by this neoplasia The nursing diagnoses identified can be used in a checklist in the patients’ medical records to aid nurses in the development of the nursing process; therefore, for every nursing diagnosis, a care plan could be elaborated. As the use of nursing diagnosis is applicable to improve nursing practice in oncology, nurses should be formally educated, prepared and encouraged to assist cancer patients giving emphasis to the diagnostic step of the nursing process.
Ogasawara *et al* [18], Japan, 2005	Descriptive (Cross-sectional) Medical Records *n* = 150 Evidence level 6	To identify nursing interventions and diagnoses in hospitalised cancer patients in terminal stage	According to NANDA’s International Taxonomy II: Chronic Pain (47.3%), Risk of Infection (43.3%), Activity intolerance (42.3%), Risk of Injury (41.3%), and Anxiety (37.2%).	The identified nursing diagnoses were elaborated under the influence of the hospitalisation reasons of the breast cancer patients in terminal stage. Therefore, oncology nurses need to consider those hospitalisation reasons when formulating nursing diagnoses and proposing intervention plans, and, they need to be aware of the socio-cultural influences that affect their attitudes and behaviours towards the patients in terminal stage.
Souza e Gorini [19], Brazil, 2006	Case studies Medical Records Physical Exams and Interviews *n* = 06 Evidence level 6	To identify nursing diagnoses in hospitalised adults with acute myeloid leukaemia	According to NANDA’s International Taxonomy II: Risk of Body Temperature Imbalance (100%), Constipation (100%), Risk of Infection (100%), Imbalanced nutrition: less than body requirements (100%), Impaired Comfort (100%), Activity Intolerance (100%), Disturbed Sleep Pattern (100%), Impaired Oral Mucous Membrane (100%), Ineffective Family Therapeutic Regimen Management (100%), Interrupted Family Processes (100%), Disturbed Body image (100%) Leisure Deficit (100%), Efficient Therapeutic Regimen Management (83.3%), Impaired Swallowing (66.6%), Anxiety (66.6%), Acute Pain (50%), Self-Care Deficit for Bathing/Hygiene, (50%), Fear (50%), Knowledge Deficit [not specified] (50%)	The 32 nursing diagnoses identified by the study were sorted by order of importance, such as at the five levels of the Maslow Hierarchy of Basic Human Needs: With the Physiological Needs – considered the immediate ones – forming the base of the pyramid with 15 nursing diagnostics and the Self-Realisation Needs, the top with 01 nursing diagnosis.
Santos *et al* [20], Brazil, 2007	Descriptive (Cross-sectional) Physical Exams and Interviews *n* = 20 Evidence level 6	To identify the more frequent emotional nursing diagnoses in the oncology preoperative visit	According to NANDA’s International Taxonomy II: Knowledge Deficit [not specified] (80%), Fear (75%), Anxiety (70%), Disturbed Sleep Pattern (70%), Interrupted Family Processes (55%), Impaired Comfort (55%), Acute Pain (50%), Disturbed Body Image (25%), Recreational Activity Deficit (20%), Risk of Relocation Stress Syndrome (20%).	Emotional nursing diagnoses and the description of the related factors enlighten the main causes to patients’ development of emotional disturbances, as such becoming an important indicator to their necessary emotional support.
Napoleão *et al* [21], Brazil, 2009	Descriptive (Cross-sectional) Physical Exams and Interviews *n* = 20 Evidence level 6	To identify nursing diagnoses in hospitalised patients who underwent prostatectomy	According to NANDA’s International Taxonomy II: Knowledge Deficit [indwelling urinary catheter and postoperative care] (100%), Risk of Fluid Volume Imbalance (100%), Risk of Injury (100%), Risk of Infection (100%), Impaired Tissue Integrity (100%), Risk of Situational Low Self-Esteem (37.5%), and Enhanced Spiritual Well-being (25%).	The investigation of nursing diagnoses among patients who underwent prostatectomy offers basis for the elaboration of nursing care plans and confirms the importance of the role of the nurse in the postoperative specific care and in the preparation of patients for hospital discharge.
Lopes *et al* [22], Brazil, 2013	Descriptive (Cross-sectional) Medical Records *n* = 185 Evidence level 6	To identify the nursing diagnoses in hospitalised women in the oncology unit in the mastectomy postoperative period, with emphasis to the psychosocial and spiritual sphere	According to NANDA’s International Taxonomy II: Risk of Infection (95.1%), Anxiety (48.6%), and Fear (41.6%).	Aspects of the Social sphere should be investigated and discussed with the mastectomised woman and her family before the hospital discharge. Therefore, the investigation of the nursing diagnoses of the Self-Perception Domain should be included in the postoperative care plan of this population The nurse is the health professional that stays in contact the longest with the hospitalised woman with breast cancer, which allows for an integral performance, supported by the nursing diagnoses, either in his/her role of care provider or of the health educator. These actions give value to the professional autonomy of the nurse and reinforce the importance of the use of the process of nursing, ensuring a systematisation of the nursing actions and their proper documentation.

**Table 2.** Most common nursing diagnoses among adults/seniors hospitalised with cancer, according to the articles included in the integrative review.

Nursing Diagnoses^*^	Article 1 [14]	Article 2 [15]	Article 3 [16]	Article 4 [17]	Article 5 [18]	Article 6 [19]	Article 7 [20]	Article 8 [21]	Article 9 [22]
**Anxiety**	-	-	-	53.3%	37.2%	66.6%	70%	-	48.6%
**Deficient Knowledge**	-	25%	-	40%	-	50%	80%	100%	-
**Constipation**	63%	-	26.6%	-	-	100%	-	-	-
**Bathing Self-Care Deficit**	56%	-	-	66.6%	-	50%	-	-	-
**Disturbed Body Image**	-	-	-	20%	-	100%	25%	-	-
**Acute/Chronic Pain**	44%	58.6%	-	80%	47.3%	50%	50%	-	-
**Fear**	-	-	-	-	-	50%	75%	-	41.6%
**Disturbed Sleep Pattern**	-	-	-	93.3%	-	100%	70%	-	-
**Risk for Infection**	63%	47.4%	100%	66.6%	43.3%	100%	-	100%	95.1%
**Risk for Deficient Fluid Volume**	-	21.9%	20%	20%	-	-	-	-	-

* Diagnoses Titles according to NANDA International, (NANDA-I), Taxonomy II [3]
